# Policymaking based on CERs: changes in costs are not the same as changes in benefits

**DOI:** 10.1186/1478-7547-3-3

**Published:** 2005-03-23

**Authors:** Andre JHA Ament, Silvia MAA Evers

**Affiliations:** 1 Department of Health Organization, Policy and Economics, University of Maastricht, Maastricht, the Netherlands

## Abstract

**Background:**

Earlier cost-effectiveness studies showed the cost-effectiveness ratios for pneumococcal vaccination in preventing cases of Bacteremic Pneumococcal Disease (BPD) alone to vary between € 11,000 and € 33,000 per quality-adjusted life year. If vaccination is also assumed to be effective in preventing cases of Non Bacteremic Pneumococcal Disease (NBPD) (at the same level of effectiveness), vaccinating all elderly persons becomes highly cost-effective or even cost saving.

**Methods:**

The present article examines the effect of partial preventive power of the vaccine against cases of NBPD additional to its preventive power against cases of BPD, and the consequences this has in terms of cost-effectiveness.

**Results:**

The analysis shows that even a fairly small additional preventive power against cases of NBPD leads to a dramatic and unexpected decrease in the cost-effectiveness ratio.

**Conclusion:**

Because a Cost-Effectiveness Ratio (CER) is a ratio, changes in costs and changes in effects have rather different influences on its value. There is a linear relation between a change in costs and a change in CER if the effects are kept constant. This linear relation is not found on the effect side. Assuming that costs are constant, a change in effect will be different for low levels of effect than for high levels.

## Introduction

Data on efficacy or effectiveness are often among the cornerstones of the calculations of Cost-Effectiveness Ratios (CERs) and information on such effect variables is crucial for the decision-making process [1,2].

As an example, this article considers the lack of information regarding the effectiveness of the vaccine for pneumococcal pneumonia. There is rather strong evidence that this vaccine prevents cases of Bacteremic Pneumococcal Disease (BPD), which however represents only a minority of cases of pneumococcal disease (about 15%) [3-7]. There is much less evidence (some experts say no evidence) that the vaccine also prevents cases of Non Bacteremic Pneumococcal Disease (NBPD) [8]. Several published studies have calculated cost-effectiveness ratios (CERs) of pneumococcal vaccination programmes for the elderly [9-14]. In the earlier studies, the assumption was made that the vaccine was able to prevent cases of NBPD to the same degree as it prevents cases of BPD. The overall conclusion of the studies based on this equal effectiveness assumption was that vaccination of the elderly is highly cost-effective, or indeed in many instances even cost saving. However, the results of these studies did not have a great impact on the use of the vaccine in many countries. The hesitation to use the vaccine can be explained by various factors, including the rigorous assumption of equal effectiveness of the vaccine for BPD and NBPD. Three more recent studies have focused on the BPD subgroup in calculating the CERs of the vaccination programme [11,13,14]. If it can be shown that the vaccine is cost-effective by preventing cases of BPD alone, this would mean that the vaccine would definitely be cost-effective if it additionally prevents some cases of NBPD, which form the majority of cases. However, the three studies yielded inconsistent results. Sisk et al. [11] presented results showing that vaccination would save costs in the US, whereas the other two studies [13,14] presented CERs for European countries ranging from € 10,000 and € 40,000 per quality-adjusted life year (QALY) for different age groups.

All of the above-mentioned studies regarded the preventive power of the vaccine against cases of NBPD as a binary variable: either the vaccine prevents no cases of NBPD or it prevents cases of NBPD to the same degree as it prevents cases of BPD. At this point, there is not much empirical evidence regarding this crucial aspect. Moreover, it is not to be expected that more specific information on the efficacy of the vaccine against NBPD will become available in the near future. Due to power rules, such a trial would cost a tremendous amount of money. This means that decisions about the introduction of the vaccine and the further expansion of its use will have to be based on present-day knowledge, with its inherent uncertainty regarding the vaccine's effectiveness.

In this article we take one further step in investigating the possible partial preventive power of the vaccine against cases of NBPD additional to its preventive power against cases of BPD, and the consequences of such additional prevention in terms of cost-effectiveness. In an earlier short article [15], we discussed the results of an additional partial effectiveness of the vaccine against cases of NBPD, and attributed the unexpected results we found merely to the fact that there are far more cases of NBPD than of BPD. Due to space limitations, we were unable to address the technical aspects of the additional analysis in that article, or to discuss the consequences in more detail. Nor were we able to explain the specific influence of the partial effectiveness of the vaccine against cases of NBPD on the resulting CERs. The present article conducts a more profound analysis and discusses the unexpected results in more detail. Additionally, it uses a graphical method to explain the results. It also explores why the results are unexpected, an analysis that might be useful for other investigators who are confronted with similar problems.

The next section provides some background information, together with the overall results of the European study (15), an analysis that forms the basis of all further analyses in this article. This is followed by a section explaining the methodological aspects of the additional analysis and presenting the detailed results of this analysis, including the influence of a potential additional preventive power against cases of NBPD. The final section further investigates why these results are unexpected and draws more general conclusions from the analysis.

## Background

The present additional analysis is based on the results of an earlier study of the cost-effectiveness of a vaccination programme in five European countries [15]. The analysis uses clinical and economic variables that are unique to each country and considers cost savings from simultaneous administration of pneumococcal and influenza vaccines. In the base case analyses, the cost-effectiveness ratios for pneumococcal vaccination in preventing cases of BPD alone vary between € 11,000 and € 33,000 per QALY among the five countries. Using more plausible epidemiological assumptions of the incidence (50 cases per 100,000) and mortality (20% to 40%) of invasive disease, the cost-effectiveness ratios are € 12,000 or less per QALY in all five countries. If vaccination is also assumed to be effective in preventing cases of NBPD (at the same level of effectiveness), vaccinating all elderly persons would be highly cost-effective or even cost saving.

The above analysis (16) included only CERs corresponding to extreme positions: either no preventive power against NBPD or the same degree of preventive power against NBPD as against BPD.

Our implicit assumption was that intermediate positions between these two extremes could be easily calculated by simple interpolation of the results in terms of CERs. If, for example, the CER for BPD alone in a particular country were 30,000 and the CER for BPD and NBPD were 10,000, we would expect, from a prior point of view, that, starting from the situation 'BPD alone' (CER = 30,000) an additional 10% preventive power against NBPD would lead to a situation characterised by a CER of (say) 28,000 (10% of the difference between the two extremes). We did not anticipate that such a small additional preventive power would in fact have a major impact on the resulting CER. Explaining this huge difference between expectations and reality requires a stepwise analysis.

## Methods

Since this additional analysis was not foreseen at the start of the above European study [15], it was not possible to apply the existing model ex post to produce the CERs for intermediate levels of effectiveness directly. However, it was possible to derive the CERs for intermediate steps of preventive power in an alternative way. Although the model developed for the European study is a rather complex and sophisticated cohort model, the outcomes of the model in terms of costs and benefits are linearly related to the input variable 'efficacy'. To calculate the costs, for example, the model starts with the incidence of the disease, after which the incidence is multiplied by the efficacy of the vaccine. This result is then multiplied by the protection rate of the vaccine, and the resulting figure is multiplied by the number of cases hospitalised. The result is then multiplied by the number of hospital days, and finally by the cost of one hospital day. This multiplicative model leads to the conclusion that the input variable 'efficacy' shows a linear relation with the outcome variable 'costs', a conclusion that is verified by the model. This linearity is also found for the outcomes in terms of QALY's. The consequence of this linear relation is that increasing the efficacy rate (expressed as a percentage) by a certain step results in the same change in effect, expressed as QALY's or as costs. In algebraic terms the cost can be expressed in the following formulas:

1) C = a + b * x (0<x<1)

2) Q = c + d * x (0<x<1)

where:

C is costs

Q is QALY's

a, b, c and d are constant

x is vaccine efficacy for cases of NBPD

The presence of linearity in the model made it possible to interpolate between the extreme outcomes (zero prevention against NBPD and same efficacy against NBPD as against BPD). As an example, let us discuss the results for Scotland in more detail.

The data for 'step 0' and 'step 10' include the existing data on the costs and benefits for Scotland, which were used for the calculation of the CERs [15]. Step 0 corresponds to the situation where the vaccine is assumed to prevent only cases of BPD, whereas step 10 assumes that the vaccine prevents cases of NBPD to the same degree as it prevents BPD. The 10 intermediate steps are interpolated, based on these two extremes.

## Results

The first surprising observation that can be made from Table 1 is that in the first step, the CER drops from an initial level of about € 15,000 per QALY to a level of € 7,000 per QALY. In the next step, where the vaccine is assumed to prevent an additional 10% of potential cases of NBPD, the CER drops to a level of about € 4,000. Alternatively, it can also be concluded in this case that an improvement in the effectiveness from say 70% to 100% would have not much impact on the resulting CER, as it would decrease from € 705 per QALY to € 242 per QALY! This makes predicting the influence of a change in effectiveness on the CER virtually impossible. The linearity that was present in the separate calculations of costs and benefits (see the first columns of the table) is no longer found in the CERs in the final column. In this specific decision environment, this would imply that if a decision maker would refuse to finance a vaccination programme based on the hard evidence for vaccine efficacy for BPD alone, a small additional preventive power of the vaccine against cases of NBPD would suffice to make it a (very) cost-effective intervention.

**Table 1:** Cost-effectiveness ratios for intermediate degrees of vaccine efficacy in preventing cases of NBPD in Scotland

Assumed preventive power for NBPD	Costs in € Million*	QALY's*	CER	Reduction in CER as percentage
0% effectiveness for NBPD	147.7	9923	14892	
Step 1 (10% increase)	135.6	19777	6858	53.9
Step 2	123.4	29630	4167	18.1
Step 3	111.3	39483	2820	9.1
Step 4	99.1	49337	2010	5.4
Step 5	87.0	59190	1470	3.6
Step 6	74.9	69044	1084	2.3
Step 7	62.7	78897	795	1.9
Step 8	50.6	88750	570	1.5
Step 9	38.4	98604	390	1.2
Same effectiveness against NBPD as against BPD	26.3	108457	242	1.0

*Cost and benefits of intermediate steps in preventive power against NBPD interpolated based on the linearity of the model

This phenomenon is by no means specific to Scotland. Table 2 shows it to be present in all five European countries, although the effect is larger in some countries than in others.

**Table 2:** Cost-effectiveness ratios for intermediate degrees of vaccine efficacy in preventing cases of NBPD in 5 European countries.

Assumed preventive Power against NBPD	Belgium	France	Scotland	Spain	Sweden
0% effectiveness against NBPD	25907	19182	14892	10511	32675
Step 1 (10% increase)	13678	15801	6858	4383	10158
Step 2 (20% increase)	8313	13085	4167	2126	4217
Step 3 (30% increase)	5300	10857	2820	954	1473
Step 4 (40% increase)	3370	8995	2010	235	Cost saving
Step 5 (50% increase)	2028	7415	1470	Cost saving	Cost saving
Step 6 (60% increase)	1041	6058	1084	Cost saving	Cost saving
Step 7 (70% increase)	285	4881	795	Cost saving	Cost saving
Step 8 (80% increase)	Cost saving	3849	570	Cost saving	Cost saving
Step 9 (90% increase)	Cost saving	2938	390	Cost saving	Cost saving
Step 10 (same effectiveness against NBPD as against BPD)	Cost saving	2127	242	Cost saving	Cost saving

From a decision analysis point of view, the changes in the first steps are particularly relevant, because they imply that a fairly small additional preventive power against NBPD makes the vaccine cost-effective. With the exception of France, the CERs in all countries decrease by 50% or more in the first step (10% preventive power against NBPD cases). In the second step (20% preventive power against NBPD) the CERs are reduced further, to even more attractive levels.

This additional analysis leads to the general conclusion that a relatively small preventive power of the vaccine against cases of NBPD is enough to make vaccination for pneumococcal disease a very attractive investment compared with other health care interventions.

## Discussion

At the start of our European study of the cost-effectiveness of the pneumococcal vaccine [15], we did not have a clue about the existence of the phenomenon described above, nor would we have thought that the impact of a small additional preventive power against NBPD would be as great as it turned out to be. This is why only the two extreme situations for the effectiveness of the vaccine were considered in our original analysis. None of the earlier articles on cost-effectiveness investigated or even mentioned this phenomenon. What is the reason for the huge differences between reality and (our) expectations at this point?

We will answer this question in two different ways: an algebraic and a graphical way.

For the algebraic analysis the formula's 1 and 2 have to be specified a little bit further.

The costs as a function of vaccine efficacy (x) can be expressed as follows:

3) C = C^0 ^- x * (C^0- ^C^1)^

where: C^0 ^is costs if x = 0

C^1 ^is costs if x = 1

C^0 ^and C^1 ^(for Scotland) can be found in table 1 and are € 147.7 million and € 26.3 million respectively.

The cost as a function of vaccine efficacy for NBPD is therefore:

4) C = 147.7 * 10^6 ^- x * 121.4 * 10^6^

The number of QALY's as a function of vaccine efficacy (x) can be expressed as follows:

5) Q = Q^0 ^- x * (Q^0-^Q^1)^

where: Q^0 ^is number of QALY's if x = 0

Q^1 ^is number of QALY's if x = 1

Q^0 ^and Q^1 ^(for Scotland) can be found in table 1 and are 9923 and 108457 respectively.

The QALY's as a function of vaccine efficacy for NBPD is therefore:

6) Q = 9923 - x * 98534

The CER as a function of vaccine efficacy for NBPD has the following form:

7) f(x) = (C^0 ^- x * (C^0- ^C^1)^)) / (Q^0 ^- x * (Q^0-^Q^1)^)), (0<x<1).

For x is 0 the value of the function becomes C^0 ^/ Q^0^, which is our original ICER for zero vaccine efficacy for NBPD. For x is 1 the value of the function becomes C^1/^Q^1^, which is our original ICER for 100% vaccine efficacy for NBPD.

It is obvious that in general de function f(x) is an exponential declining function, which explains, to a certain degree, the specific behaviour of the CER; huge declines in CER for low values of x and small declines when x reaches the value 1. An impression of this can be based on the data of table 1, where the CER's (of Scotland) as a function of vaccine efficacy for NBPD are given in column 4.

**Figure 1:**
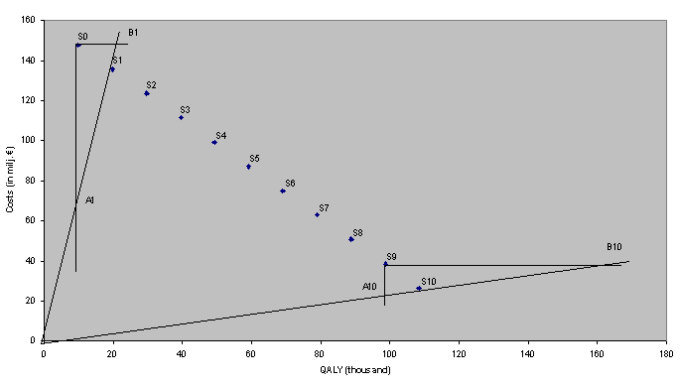
Costs and benefits of intermediate steps of partial effectiveness against NBPD for Scotland

A graphical representation allows us to clarify the causes of this phenomenon in another way. Figure 1 shows a graphic representation of the data for Scotland. One extreme represents the costs and benefits under the assumption that the vaccine prevents only cases of BPD (S_0_) while the other extreme represents the assumption that cases of NBPD are prevented to the same degree as those of BPD (S_10_). The other points represent the costs and benefits in the consecutive steps. Due to the linearity of the model, these 10 steps are positioned on a straight line, and indicated as S_1_, S_2 _etc.

But why do the CERs for S_0 _to S_10 _show non-linear behaviour?

We have to show that equal steps in costs and/or benefits do not automatically lead to equal differences in CERs. The first step (10% preventive power against NBPD) takes us to point S_1_. Starting from S_0_, there are numerous alternative ways in which a CER of this level could be reached. One of these ways would be to lower the cost from S_0 _to level A_1 _(see figure), another to increase the benefits from S_0 _to B_1_, but other combinations are also possible. A_1_, S_1 _and B_1 _are on the same line through the origin and therefore have the same CER.

Starting from S_0_, a rather small increase in benefits has the same effect (in terms of CER) as a rather large decrease in costs. Looking at the steps from S_9 _to S_10_, we see the opposite: in this area a rather small decrease in costs has the same impact as a rather huge increase in benefits. It will be clear from this observation that the ratio between costs and benefits changes in each of the 10 steps. This phenomenon can be visualised in a different way, using the structure of CERs.

As many authors have suggested, CERs can be visualised in graphic representations [[1,2,16-18], and [19]]. These graphs may help to identify the most cost-effective interventions within a set of compatible (mutually non-exclusive) alternatives. Graphs can also be helpful in identifying the best candidate in a set of incompatible (mutually exclusive) alternatives [17]. Often there is no need to pay attention to the precise underlying structure of CERs. For exemple, Laupacis et al. [16] in their original article drew CER lines in a rather arbitrary way.

The above observations do not hamper decision-making, as long as only 'ordinal' conclusions are drawn, for example, that intervention A is more cost-effective than intervention B. However, absolute changes in the position of an intervention in the graph might lead to unexpected results, as will be shown below.

**Figure 2:**
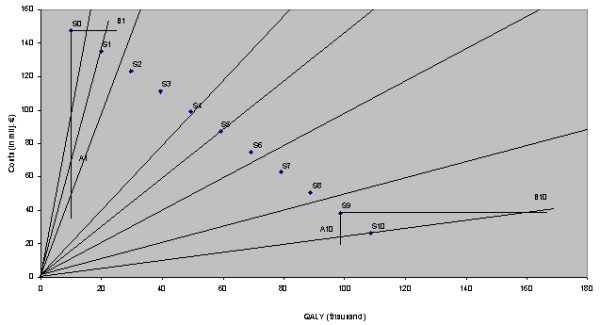
CERs for technologies characterised by costs per QALY of € 1,000, € 2,000, € 3,000 etc. can be easily constructed using the constant vertical distance (a) between the lines

In figure 2 the exact position of different CER-lines are drawn, based on figure 1. The figure clearly shows that different incremental increases in preventive power of the vaccine must lead to different effects on the resulting CER. The first increase in preventive power against cases of NBPD (step 1) cuts from a CER of (about) € 15,000 per QALY through a whole series of nearby CER lines and ends up at a CER of € 7,000 per QALY. The next step would bring this down further, to a CER of € 4,000, a relatively minor decrease compared with the previous step. In absolute terms, however, both steps are identical, in that they consist of the same decrease in costs and benefit.

The above analysis allows the conclusion that a particular change in costs and benefits will not lead to a fixed change in the CER. The impact of such a change in costs and/or benefits on the resulting CER depends on the starting point in the graph. In our example, it could be said that the position of the line S0-S10 determines the resulting CER. This is the reason why the effects of a partial effectiveness of the pneumococcal vaccine against NBPD are different in different countries.

## Conclusion

If the pneumococcal vaccine has even a small preventive power against NBPD, this would make vaccination against pneumococcal pneumonia a very cost-effective intervention. While it is very unlikely that the vaccine is as effective in preventing cases of NBPD as it is in preventing cases of BPD, it is also unlikely that the vaccine does not prevent any cases of NBPD. The above analysis shows that a very small additional preventive power against NBPD (about 5 to 10%) changes the cost-effectiveness of this vaccination programme, on average, from a level just below the maximum value per QALY (€ 20,000 per QALY) to a level of € 10,000 per QALY, a level that in most industrialised countries would be considered a good health investment. Without the above analysis, policy-making would be very complicated, because of the uncertainty about the preventive power and the uncertainty about the maximum value per QALY. With this analysis, there is less uncertainty and policymakers can be more confident in deciding to introduce the vaccination programme for the population aged 65 and older.

Is there a more general principle behind the above phenomenon?

To answer this question we have to look back to the concept of CER itself. CER is defined as (incremental) costs divided by (incremental) effects. It is obvious that a CER increases as costs increase, if the effects are kept constant. An increase in cost by an amount x will lead to an increase in CER of y, independent of the volume of the costs. There is a linear relation between a change in costs and a change in CER if we keep the effects constant.

This linear relation is not found on the effect side. Assuming that the costs are constant, a change in effect will be different for low effect levels than for high levels. Adding 1 QALY to an intervention that produces 1 QALY leads to a reduction of 50% in CER, whereas adding 1 QALY to 10 QALY's results in a 10% reduction in CER. With regard to effects, a *relative *increase will always lead to the same change in CER, whereas with regard to the costs, an *absolute *change will lead to the same change in CER. In the above case of a partial increase in preventive power, the costs and benefits increase by the same amount in each step, but we now see that it is merely the effects that lead to the unexpected results: merely changing the costs by a particular amount would not have this effect. We stated above that our model was a multiplicative model as regards costs and effects. We used this property of the model only to calculate costs and benefits corresponding to intermediate steps in preventive power in an easy manner. We can therefore conclude that the above results are not restricted to this type of linear model, but are to be expected in general. The above phenomenon depends on the ratio approach, not on the model used.

The above analysis leads to several observations In the first place, intuition might be not a good instrument to predict the CER consequences of changes in costs and (especially) benefits. In the above example the consequences of partial effectiveness of the pneumococcal vaccine for the CERs were totally unexpected. In the example, the effectiveness determines both costs and benefits of the CER, which makes prediction of effects more complicated. Researchers who are thinking of performing sensitivity analyses should be aware of the fact that in some cases even very small changes in variables could have huge impacts on the resulting CERs. It will not always be easy to anticipate or predict this impact, due to the exponential character of the CER itself.

Laupacis et al. (1992) were among the first to use graphs to represent CERs of different interventions, although the principle had already been described as early as 1986 (See for example Anderson et al., 1986). Gold et al. suggested that researchers should make use of such graphs, because they are handsome, concise and easy to understand. In their original graph, Laupacis et al. represented two CERs: one for interventions with a CER of $20,000 and the other with a CER of $100,000. The two lines in their figure are drawn rather arbitrarily, whereas in reality the position of the two lines should be fixed (see above). In analysing the results in a graph, one should understand the underlying structure of the CERs (see figure 2), especially in cases where interventions are presented in absolute positions, as was the case in our example of the pneumococcal vaccine. If the graph is used to prioritise compatible alternatives according to cost-effectiveness, using the wrong graph would be no big problem. However, if one is interested in identifying the best candidate within a set of incompatible alternatives, using the right framework becomes crucial.

A last observation of our analysis has to do with the term 'cost-effective' itself. It has already been recognised before that the term 'cost-effective' is rather unspecified. What is the real meaning of the expression: intervention A is more cost-effective than intervention B? This becomes even more troublesome if one considers different interventions in a QALY League Table. People are often inclined to think that the difference between interventions with CERs of 10,000 and 20,000 is the same as that between interventions with CERs of 50,000 and 60,000, because the difference in CER is the same in both situations. In reality, it is impossible to decide which of the two interventions is better as long as we have no separate information on costs and benefits. This observation also leads to the conclusion that it is impossible to say whether it is better to improve the cost-effectiveness of one particular technology from (for example) € 10,000 to € 20,000 than to improve the cost-effectiveness of another technology from € 50,000 to € 60,000. This means that the cost-effectiveness scale in itself is not a cardinal scale but only an ordinal scale. Needless to say, the incremental-net-benefit parameter recently proposed by many authors behaves in a much more straightforward way in this respect than the above incremental CER, providing another argument in favour of the net benefit approach.

## Competing interests

The original study was supported by a grant of Aventis Pasteur MSD. The above study was performed totally independent from Aventis Pasteur MSD.

## Authors' contributions

The authors worked together in close cooperation on the concepts and methodological aspects of the study. The first author produced the first drafts, whereas the second author produced many valuable contributions.
